# Cross-Sectional Associations between Empirically-Derived Dietary Patterns and Indicators of Disease Risk among University Students

**DOI:** 10.3390/nu8010003

**Published:** 2015-12-24

**Authors:** Stacy A. Blondin, Megan P. Mueller, Peter J. Bakun, Silvina F. Choumenkovitch, Katherine L. Tucker, Christina D. Economos

**Affiliations:** 1 Tufts University Friedman School of Nutrition Science and Policy, 150 Harrison Avenue, Boston, MA 02111, USA; m.mueller@tufts.edu (M.P.M.); peter.bakun@tufts.edu (P.J.B.); silvina.choumenkovitch@tufts.edu (S.F.C.); christina.economos@tufts.edu (C.D.E.); 2 Clinical Laboratory & Nutritional Sciences, Center for Population Health & Health Disparities, University of Massachusetts at Lowell, 3 Solomont Way, Suite 4, Lowell, MA 01854, USA; katherine_tucker@uml.edu

**Keywords:** dietary patterns, principle component analysis, college students, BMI, percent body fat, blood lipids, Tufts Longitudinal Health Study

## Abstract

The transition from adolescence to adulthood is a unique period during which lifelong dietary habits are shaped. Dietary patterns (DPs) among young adults attending college have not been adequately described, and associations between DPs and indicators of disease risk are not well understood in this age group. Dietary data were collected from undergraduates participating in the Tufts Longitudinal Health Study (TLHS; 1998–2007) by Food Frequency Questionnaire (FFQ; *n* = 1323). DPs were derived using principal components analysis with varimax rotation. Scree plots; eigenvalues; factor loadings; and previous studies were used to determine and label the DPs retained. Cross-sectional relationships between DP scores and anthropometric measures (percent body fat (PBF) and (BMI) and lipid biomarkers (total; HDL and LDL cholesterol; and triglycerides) were assessed with multivariable regression models; adjusted for demographics; physical activity; smoking; intention to gain/lose weight; and total energy intake. Effect modification by sex was tested. Three DPs were identified: Prudent; Western; and Alcohol. Greater adherence to the Prudent DP was associated with favorable anthropometric outcomes. The Alcohol DP was associated with a favorable lipid profile. Associations between the Western DP and blood lipids differed by sex; with unfavorable impact observed only among males. Our findings add to the literature linking DPs in young adults with measurable adiposity and cardiometabolic outcomes; suggesting that improving nutrition among college students could reduce chronic disease risk.

## 1. Introduction

Dietary composition represents the largest risk factor for mortality and disease burden in the United States, responsible for 26% of deaths and 14% of disability-adjusted life years (Murray *et al.*, 2013). The most important dietary risks in the United States include low intakes of fruit, vegetables, nuts and seeds and high intakes of sodium, added sugars, processed meats, and *trans* fats. Because dietary intake is a modifiable lifestyle behavior, interventions to help individuals make positive dietary choices throughout the lifespan have the potential to substantially improve the quality and duration of life.

The transition between adolescence and adulthood, characterized by increasing independence, autonomy, and responsibility, is often the first time period in which individuals make autonomous decisions about “how, what, where, and when to eat” [1] and is, therefore, a crucial life-stage for establishing life-long health behaviors and habits, including healthy eating patterns [2]. For many, the transition into adulthood results in a shift in composition and quality of the diet. In fact, most studies have found that diet quality may worsen during this transition [1]. Data from the 2005–2006 and 2003–2004 NHANES suggest that the 13–18 and 19–39 year age groups consume the most caloric beverages [3] and are most likely to fall short of meeting fruit and vegetable serving recommendations [4]. Longitudinal studies support these findings, showing that consumption of fruit, vegetables, and milk generally decreases, while intake of sweetened beverages and snack foods tends to increase [5,6,7]. The transition to young adulthood has also been associated with increased frequency of fast food consumption and reduced frequency of breakfast consumption [8,9].

An increasing proportion of young adults are attending colleges and universities in the US, such that 20.2 million are expected to enroll in a degree-granting program in 2015, up by 4.9 million since 2000 (15% increase 1992–2002, 24% increase 2002–2012). With more than half of the college-going population attending four-year universities (13.2 million) and most enrolling full time (12.6 million) [10], the college population is of particular interest as a target group for health promotion- and disease-prevention interventions. To date, the majority of studies on diet-related health outcomes among college students have focused on weight status and/or trajectory, especially weight gain during the transition period from home to college during the freshman year [11,12,13,14].

A recent meta-analysis of studies from 1960 to 2013 (*n* = 49) on weight gain during the college years reported adjusted mean effect sizes of 1.55 kg increase in body weight and 1.17% increase in percent body fat (PBF) [12]. Others have explored potential factors that predict weight gain during college, such as place of residency, physical activity, psycho-social and sociodemographic factors, and body image/self-perception [15,16,17,18,19,20,21,22,23,24]. Though dietary behavior and intake among college students have been topics of longstanding interest [25,26,27,28,29,30,31,32,33,34], the extent to which variability in dietary patterns contribute to weight status among first-year college students has received limited attention. Most studies have investigated whether college students meet dietary guideline recommendations and/or whether specific foods or food groups influence weight status. However, considering individual dietary components in isolation provides a limited picture of how synergies among foods and nutrients in combination (*i.e*., the diet as a whole) influence health outcomes [95].

Given the relative dearth of literature examining associations between dietary patterns and health outcomes among college students, the aim of this analysis was to investigate cross-sectional associations between empirically-derived dietary patterns and anthropometric and biomarker disease indicators among four-year university students. Identifying lifestyle factors that influence college students’ health, including dietary patterns, could inform policies, programs, and interventions designed to reduce lifelong chronic disease risk of current and future generations. Although this life stage has been recognized as a critical time period for establishing nutrition behaviors carried into adulthood [2], and dietary patterns have been recognized as an important determinant of health [35,36], additional research is needed to elucidate relationships between the two.

## 2. Materials and Methods

### 2.1. Sample and Study Design

The Tufts Longitudinal Health Study (TLHS) is a prospective cohort study on the health and health-related behaviors of undergraduate students at Tufts University, a private research university in Medford, MA. All undergraduate students enrolling from 1998 to 2007 were eligible and recruited to participate. Members of each incoming freshmen class received a soft and hard version of the study’s informed consent form, with information about the objectives, potential risks and benefits, and procedures in late July/early August, prior to the start of the academic year. Students who read, signed, and returned the form were enrolled in the study. Participants completed a 40-item Health Behavior Survey (HBS) before arriving at school and were invited to participate in a follow-up health assessment each spring. During the spring health assessment, students completed the HBS, a Food Frequency Questionnaire (FFQ), anthropometric and physical fitness measurements, and an optional blood draw. These assessments took place in April (approximately eight months after baseline HBS administration) and were repeated freshman through senior year. All procedures were performed in accordance with human subject research standards and approved by the Tufts University Institutional Review Board.

**Figure 1:**
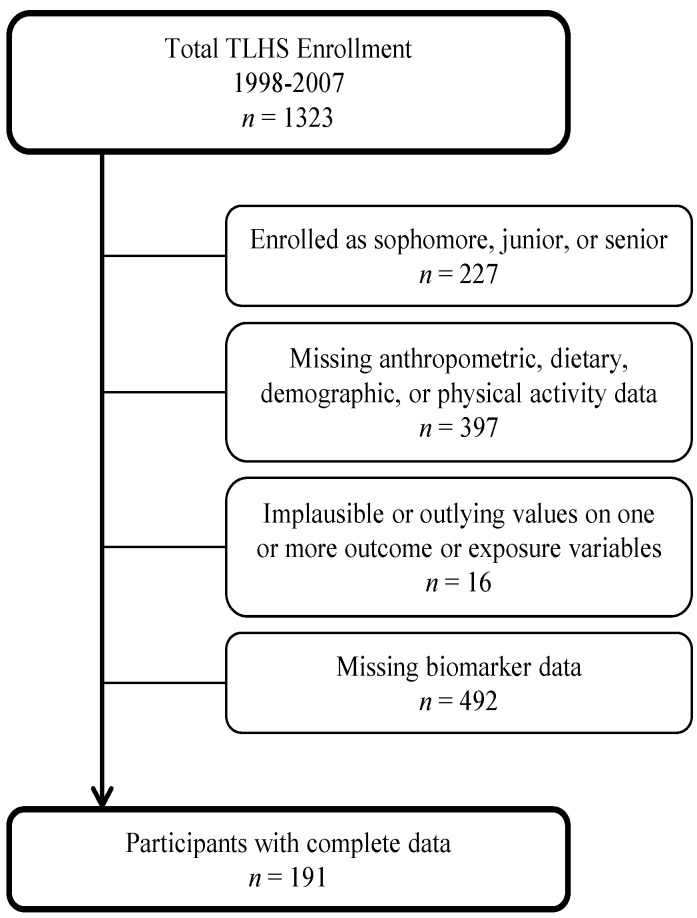
Flow chart depicting analytic sample deduction (boxes to the right indicate the number of participants excluded for each reason).

For this analysis, we restricted the sample to students who completed the health assessment during freshman year between 1998 and 2007, which included the largest number of participants (*n* = 1096). Approximately 99.3% of these students lived on campus and all were required to purchase a campus dining hall meal plan (97.2% reported eating in the dining hall frequently). As only a subset of participants completed blood assessments, we only excluded those with missing anthropometric data and/or missing data on diet, demographics, or physical activity (*n* = 313). We excluded data from students entering college in 2003 (*n* = 84) due to inconsistencies in the data. Individuals with negative, outlying, and/or implausible values for exposure or outcome variables based on age/sex appropriate population distributions were excluded (*n* = 16). In total, 683 participants had complete information on measures of body fatness, dietary data, and covariates of interest and 191 had complete blood lipid data, dietary data, and covariates of interest (Figure 1).

### 2.2. Assessments

#### 2.2.1. Outcome Variables: Health Indices

##### Anthropometric Measurements

Anthropometric variables of interest included weight, height, body mass index (BMI), and body composition. BMI was calculated as weight (kg)/height (m^2^) from measured height and weight (collected from the spring assessment). Participants removed their shoes and wore light clothing for measurement procedures. Weight was measured using a portable balance beam scale (Healthometer, Boca Raton, FL, USA) and recorded to the nearest 1/4 pound. Height was measured using a portable stadiometer (Model 214, Seca Weighing and Measuring Systems, Hanover, MD, Germany) and recorded to the nearest 1/8 inch. Agreement between measured and self-reported weight and height was strong (*r* = 0.997, *p* < 0.001 for weight and *r* = 0.957, *p* < 0.001 for height) [37].

Body composition was determined via Bioelectrical Impedance Analysis (BIA). Total body resistance (ohms) and reactance (ohms) were measured on participants lying supine, with four surface self-adhesive spot electrodes and a standard conduction current of 800 lA and 50 kHz (BIA Model 101, RJL Systems, Detroit, MI, USA) following standard procedures. The laboratory-reported intra-operator (*n* = 4 operators; *n* = 4 subjects; triplicate measures) CV was 2.15% for resistance and 3.70% for reactance, and the inter-operator (*n* = 6 participants; 2–3 measures each) CV was 2.70% for resistance and 3.25% for reactance.

Fat Free Mass (FFM) was calculated using the race-combined equations developed by Sun *et al.*, (2003) Equation (1) for men and women, and a two compartment model was used to calculate total body fat (BF) and PBF by subtracting FFM from total mass [38].

FFM_Males_ = _9.88 + 0.65 stature^2^/resistance + 0.26* weight + 0.02* resistance
FFM_Females_ = _11.03 + 0.70 stature^2^/resistance + 0.17* weight + 0.02* resistance (1)

##### Lipid Biomarkers

Blood samples were collected in 2000–2002, 2004, 2006, and 2007. Blood was drawn after a 12-h overnight fast. Serum lipid profiles (total cholesterol, HDL cholesterol, and triglycerides) and glucose were measured by ACEi Clinical Chemistry Systems (Schiapparelli Biosystems, Fairfield, NJ, USA) using standard reagent kits. LDL cholesterol was calculated via the Friedewald equation [39].

#### 2.2.2. Exposure/Predictor Variables: Dietary Intake and Dietary Patterns

Dietary data were collected using an adapted version of the Fred Hutchinson Cancer Research Center Food Frequency Questionnaire (Version 06.10.88, Cancer Prevention Research Program, Fred Hutchinson Cancer Research Center, Seattle, WA, USA). Participants were asked to report how frequently they consumed each food item listed (ranging from 2 or more per day to never/less than once per month) in each of 8 main food categories (fruit/juices, breakfast, vegetables, meat/fish/poultry, breads/snacks/spreads, dairy, sweets, and beverages). They were also asked to indicate the amount of each food they usually consumed (small, medium, or large) relative to a specified medium serving size (e.g., ½ cup). FFQs were scanned using a Scantron OpScan6 optical mark reader and linked to the Minnesota Nutrition Data System (NDS) [40] for food grouping, serving size, and nutrient intake using a SAS program maintained by the Epidemiology and Dietary Assessment Research Program, USDA Human Nutrition Research Center on Aging at Tufts University, Boston, MA, USA.

The FFQ food items (~100) were condensed into 43 food groups, adapted from Hu *et al.* (1999) [41] (Table S1). For each observation, the number of medium serving sizes of foods within each group were summed across all relevant food items. Dietary patterns were derived using principal components analysis (PCA). The resulting scree plot was used to visually assess the variability captured by each factor (Figure S1). Eigenvalues, factor loading values, and previous studies were used to determine and label the dietary patterns. Three factors with eigenvalues >2 were identified and then orthogonally rotated (varimax rotation was applied to obtain uncorrelated factors). Food groups in each factor with loadings ≥0.3 in absolute value were used to interpret the factors. Each participant received a score for each dietary pattern.

#### 2.2.3. Confounding and Effect Modifying Variables

##### Physical Activity

Self-reported physical activity and exercise habits were measured using the Cooper Institute for Aerobics Research Aerobics Center Longitudinal Study (ACLS) questionnaire [42,43,44]. For each activity included on the questionnaire (e.g., walking, stair climbing, jogging/running, bicycling, swimming, weight training, *etc.*), participants were asked to self-report the number of sessions they engaged in, the average duration of each session, and the distance and/or speed traveled in each session (as relevant), over the past three months for activities engaged in at least once a week.

Total Metabolic Equivalent of Task (MET) minutes were calculated for each student from physical activity information collected via the ACLS. The number of sessions per week was multiplied by the average session duration to calculate the total number of minutes engaged in each activity per week, and distance was divided by the duration of each session to calculate average speed for endurance activities (e.g., running, swimming, biking, walking, *etc.*). The total number of minutes engaged in each activity at each intensity value (as available/applicable) was then multiplied by the corresponding MET value [45]. When there was not an exact match in the MET compendium for an ACLS activity, we used the MET value for the most closely related activity or took an average of the MET values for the most closely related category of activities. Because a wide variety of activities were listed for the “other” category (e.g., singing *versus* rowing), we used the average MET value for all of the activities included in the compendium.

##### Total Energy

Total energy intake per day was calculated from the FFQ data by summing the total kJ per person per day across all food groups.

##### Demographic Variables

The HBS was developed from existing population-based surveys and pilot tested with more than 100 college-age students in 1998 and 1999. Questions were adapted and compiled from validated instruments and questionnaires previously used in large studies of college age students, including the National College Health Risk Behavior Survey (NCHRBS), American College Health Association (ACHA) National College Health Assessment, Youth Risk Behavior Surveillance System (YRBSS), and Colorado State University Wellness Lifestyle Profile [46,47,48]. Students self-reported their height/weight and answered questions about age, sex, and race/ethnicity.

Age was coded as a continuous variable, sex dichotomized into male and female, and self-reported race/ethnicity responses collapsed from Asian/Pacific American, Black/African American, Hispanic/Latin American, White/Caucasian, Bi-racial/Multiracial, Native American, Other into five categories: Caucasian, African American, Hispanic, Asian, and Other. Smoking status was dichotomized into smokers and non-smokers, based on self-report of current smoking behavior. Participants’ intention to gain or lose weight were dichotomous variables based on yes/no survey question responses.

### 2.3. Statistical Analysis

All analyses were performed with SAS statistical software (Version 9.3, Cary, NC, USA). *p* values < 0.05 were considered statistically significant. Distributions identified as non-normal based on the Shapiro-Wilk test for normality [49] were log-transformed (BMI, LDL, triglycerides, and total cholesterol). When log transforming non-normally-distributed outcome variables did not affect coefficient directionality or model significance, non-logged estimates were reported to enhance interpretability of results.

#### Regression Models

Multivariable regression models were used to examine cross-sectional relationships between empirically derived dietary pattern scores and BMI and lipid biomarkers. Models were adjusted for age, sex, race, physical activity (MET minutes), and total energy intake (kJ/day). KJ values <2510.4 and >20,920 were excluded, based on approximations from previous literature [50,51]. Tukey’s test was used to identify outliers for all predictor and outcome variables [52]. Outlying values for physical activity were included because they were plausible and their inclusion did not affect the directionality or significance of the results. Sensitivity analyses were run with physical activity quantified as total minutes of activity per week and with dietary pattern score categorized into quartiles. Sex was evaluated as an effect modifier; main effects were reported when no significant interaction was present. All biomarkers (HDL, LDL, and total cholesterol, and triglycerides) were treated as continuous variables.

## 3. Results

### 3.1. Sample Demographics

The analytic sample included 683 participants with HBS and biomarker data collected during their freshman year at Tufts University. Sample demographics are presented in Table 1. Briefly, participants were predominantly non-Hispanic white (76.1%), and the majority were female (68.1%). The mean age was 18.5 ± 6.0 years and ranged from 17 to 21 years. Participants were generally healthy (mean anthropometric and biomarkers within normal ranges), non-smoking, and physically active.

**Table 1:** Overall sample characteristics (*n* = 683).

Characteristic	
Male (%)	31.9
Age years	18.5 ± 0.6
Race/Ethnicity	
Caucasian (%)	76.1
African American (%)	3.4
Hispanic (%)	3.7
Other (%)	16.8
Current smokers (%)	4.1
Freshman (%)	100.0
Intention to lose weight (%)	52.3
Intention to gain weight (%)	11.2
MET minutes PA per week	2324.4 ± 2276.6
Total Daily Energy Intake (kJ)	7824.5 ± 3172.3
Percent Body Fat (%)	24.3 ± 6.9
BMI (kg/m^2^)	22.8 ± 3.0
Lipid Profile (*n* = 191)	
LDL (mg/dL)	93.8 ± 27.9
HDL (mg/dL)	54.0 ± 12.0
Total cholesterol (mg/dL)	167.0 ± 32.6
Triglycerides (mg/dL)	96.3 ± 42.1

All values are mean ± sd unless otherwise noted.

### 3.2. Dietary Pattern Characterization

Three dietary patterns were identified and labeled: Western, Prudent, and Alcohol. These patterns explained 10.3%, 9.3% and 4.8% of total variance, respectively. Food groups with PCA factor loading values ≥0.3 are listed for each dietary pattern in Table 2. Negative values reflect inverse correlations (e.g., legumes and other vegetables for the Alcohol dietary pattern). The highest values (absolute values >0.5) were observed for red meat, French fries, refined grains, processed meats, and snacks for the Western dietary pattern; fruit, vegetables (including subtypes), and whole grains for the Prudent pattern; and liquor and beer, for the Alcohol dietary pattern. In general the Western pattern was characterized by refined and energy-dense foods high in fat and sugar; the Prudent pattern by whole, plant-based foods and healthy fats and oils; and the Alcohol pattern by caloric beverages, especially those containing alcohol and, to a lesser extent, coffee (mean intake 4.68 fl oz per day among those in the highest quartile). The average medium serving sizes consumed by participants in the lowest and highest quartile of dietary pattern scores for the top five foods in each of the three patterns are listed in Table 3.

### 3.3. Dietary Patterns and Indicators of Disease Risk

Adherence to the Prudent dietary pattern was inversely associated with percent body fat and BMI after adjustment for biological and behavioral variables (Table 4). The crude, positive association between the Prudent dietary pattern and HDL was no longer significant after further adjustment. Significant dietary pattern score by sex interaction terms were observed for the Western dietary pattern and selected health indices (Table 4 and stratified models in Table 5). In crude models, the Western dietary pattern was positively associated with PBF among males and with HDL among females, although significance was reduced in adjusted models.

In general, associations between the Western dietary pattern and disease indicators were stronger and more adverse among males. The Western dietary pattern was positively associated with triglycerides among males in all three models. Although significant positive associations with LDL cholesterol and triglycerides among males were attenuated with the inclusion of lifestyle variables in Model 3, positive borderline significant associations (*p* < 0.1) were observed between Western dietary pattern and LDL cholesterol, total cholesterol, and triglycerides (Table 5). When looking at differences in food group consumption among males and females with scores in the upper quartile of Western dietary pattern, the most statistically significant differences (*p* < 0.001) were observed for beer, high energy drinks, processed meats, red meat, pizza, and sweets/desserts (Table S2). The greatest absolute differences (≥0.25 servings/day) were observed for fruit juice, sweets/desserts, beer, processed meats, and red meat. Of the differences with the greatest statistical significance and absolute value, the processed meat and red meat food groups had the highest factor loadings for the Western dietary pattern (>0.5), followed by sweets/desserts (0.31). Differences in pizza consumption were also notable, with an absolute difference of 0.16 servings per day. Though the difference in beer servings consumed was considerable (0.25 per day), the factor loading was relatively low (0.14).

Alcohol dietary pattern adherence was positively associated with HDL cholesterol and inversely associated with LDL cholesterol. These relationships were strengthened with adjustment for additional variables. Borderline positive associations were observed for the Alcohol dietary pattern and PBF in Model 1 and BMI in Model 2.

**Table 2:** Factor loading matrix for the three major dietary patterns identified from the Food Frequency Questionnaire (FFQ).

Dietary Pattern					
*Western*		*Prudent*		*Alcohol*	
Foods or Food Groups	Factor Loading	Foods or Food Groups	Factor Loading	Foods or Food Groups	Factor Loading
Red meat	0.66	Fruit	0.74	Liquor	0.55
French fries	0.59	Dark yellow-orange vegetables	0.60	Beer	0.48
Refined grains	0.58	Other vegetables	0.57	Wine	0.46
Processed meats	0.56	Whole grains	0.55	Coffee	0.38
Snacks	0.51	Cruciferous vegetables	0.52	Low-energy drinks	0.30
Potatoes	0.49	Green leafy vegetables	0.51	Legumes	−0.38
Pizza	0.48	Legumes	0.51	Other vegetables	−0.40
Butter	0.45	Non-cream soups	0.47		
High energy drinks	0.45	Tomatoes	0.44		
Pasta	0.45	Yogurt	0.43		
Creamy dressings	0.42	Nuts	0.38		
High fat dairy products	0.42	Breakfast cereal	0.35		
Ice cream	0.42	Fish and seafood	0.34		
Poultry	0.42				
Margarine	0.38				
Other fats and oils	0.38				
Fruit juice	0.37				
Sweets and desserts	0.31				

**Table 3:** Mean daily intake of participants with scores in the top and bottom quartile (Q4 and Q1, respectively) of the three dietary patterns for the foods/food groups with the highest factor loadings.

Dietary Pattern								
*Western*			*Prudent*			*Alcohol*		
Foods/Food Groups	Daily Intake		Foods/Food Groups	Daily Intake (Cups)		Food/Food Groups	Daily Intake (fl oz)	
	Q 1	Q 4		Q 1	Q 4		Q 1	Q 4
Red meat (ounces)	0.25	2.64	Fruit ^3^	0.25	1.31	Liquor	0.06	0.63
French fries (cups)	0.07	0.68	Dark yellow-orange vegetables ^4^	0.04	0.27	Beer	0.00	5.4
Refined grains (pieces) ^1^	0.56	1.89	Other vegetables	0.04	0.29	Wine	0.05	0.40
Processed meats (pieces) ^2^	0.12	1.02	whole grains ^5^	0.11	0.52	Coffee	0.36	4.68
Snacks (cups)	0.07	0.33	Cruciferous vegetables	0.03	0.25	Low-energy drinks	0.72	7.44

Mean daily intake was calculated by multiplying the mean servings consumed per day from each food group by the mean serving size (the most common serving size unit); ^1^ Serving sizes of food items in the refined grains group included 1 piece, 1 medium, 2 each, 6 small or 3 large, 1–4 ¼” diameter pastry, 1 large. The most common serving size listed was 1 piece; ^2^ Processed meats servings sizes were determined based on the serving sizes for bacon and pork sausage links: 2 links or pieces; ^3^ Serving sizes of food items in the fruit group included 1 medium, ½ cup, 1 each, ¼ cup, 3 each, ¼ of 5”diameter, ½ of 10”diameter, ½ of 4” diameter. The most common serving size listed was ½ cup; ^4^ Serving sizes of food items in the dark yellow-orange group included ½ cup and 1 medium. The most common serving size listed was ½ cup; ^5^ Serving sizes of food items in the whole grains group included 2 slices, 6 small, ½ cup, ¾ cup, 1 cup. The most common serving sizes listed were in cups, so the items with servings listed in cups were averaged.

**Table 4:** Associations between dietary pattern scores and anthropometric and biomarker outcomes.

		Model 1			Model 2			Model 3	
	β	se	p	β	se	p	β	se	p
**Outcomes**	**Western**								
Body fat (%)	(p-interaction = 0.0426)			−0.08	0.37	0.818	0.31	0.36	0.381
BMI (kg/m^2^)	(p-interaction = 0.0247)			−0.10	0.21	0.640	0.21	0.20	0.305
HDL (mg/dL)	(p-interaction = 0.0128)			−1.89	1.58	0.234	−1.55	1.60	0.336
LDL(mg/dL)	(p-interaction = 0.0460)			(p-interaction = 0.0134)			(p-interaction = 0.0148)		
Triglycerides(mg/dL)	(p-interaction = 0.0210)			(p-interaction = 0.0190)			(p-interaction = 0.0139)		
Total Cholesterol(mg/dL)	1.40	2.46	0.569	(p-interaction = 0.0271)			(p-interaction = 0.0303)		
	**Prudent**								
Body fat (%)	0.36	0.26	0.176	−0.26	0.22	0.236	−0.42	0.21	0.046 *
BMI (kg/m^2^)	−0.18	0.11	0.114	−0.15	0.12	0.217	−0.29	0.12	0.017 *
HDL (mg/dL)	2.17	0.88	0.014 *	1.29	0.92	0.165	1.19	0.96	0.216
LDL(mg/dL)	1.48	2.07	0.476	1.03	2.34	0.660	0.79	2.47	0.748
Triglycerides(mg/dL)	0.18	3.13	0.954	−1.88	3.48	0.590	−1.96	3.55	0.581
Total Cholesterol(mg/dL)	3.70	2.41	0.126	1.95	2.68	0.467	1.60	2.82	0.572
	**Alcohol**								
Body fat (%)	0.46	0.27	0.083 ^+^	0.33	0.22	0.130	0.15	0.21	0.482
BMI (kg/m^2^)	0.19	0.12	0.105	0.24	0.12	0.052 ^+^	0.13	0.12	0.280
HDL (mg/dL)	2.49	0.92	0.007 *	1.94	0.92	0.036 *	2.16	0.93	0.021 *
LDL(mg/dL)	−4.07	2.16	0.061 ^+^	−5.43	2.31	0.020 *	−5.46	2.38	0.023 *
Triglycerides (mg/dL)	1.39	3.29	0.673	−0.33	3.49	0.924	−0.59	3.48	0.866
Total Cholesterol (mg/dL)	−1.29	2.55	0.612	−3.55	2.67	0.186	−3.40	2.75	0.218

Model 1: unadjusted; Model 2: adjusted for age, race, sex, energy; Model 3: adjusted for age, race, sex, energy, smoking status, physical activity, and weight gain/loss intentions; * *p* < 0.05, ^+^ *p* < 0.1; Interaction term *p* values are reported for models with significant modification by gender.

**Table 5:** Western dietary pattern regression models stratified by sex (β (standard error), *p*-value).

	% Body Fat	BMI	HDL	LDL	Triglycerides	Total Cholesterol
**Model 1**						
Males	0.58 (0.35), *p* = 0.1	0.21 (0.21), *p* = 0.30	−0.72 (1.31), *p* = 0.58	8.34 (3.4), *p* = 0.02 *	14.1 (4.54), *p* = 0.003 *	NA
Females	−0.21 (0.30), *p* = 0.5	−0.21 (0.17), *p* = 0.21	2.48 (1.37), *p* = 0.07 ^+^	−2.58 (3.28), *p* = 0.43	−2.43 (5.22), *p* = 0.64	NA
**Model 2**						
Males	NA	NA	NA	13.0 (6.55), *p* = 0.05 ^+^	24.7 (8.59), *p* = 0.01 *	14.5 (6.99), *p* = 0.04 *
Females	NA	NA	NA	−0.84 (5.26), *p* = 0.87	−1.83 (8.14), *p* = 0.82	−1.08 (6.24), *p* = 0.86
**Model 3**						
Males	NA	NA	NA	12.2 (6.75), *p* = 0.08 ^+^	23.7 (8.19), *p* = 0.005 *	13.98 (7.22), *p* = 0.06 ^+^
Females	NA	NA	NA	−1.38 (5.53), *p* = 0.80	−4.98 (8.28), *p* = 0.55	−1.88(6.54), *p* = 0.77

Model 1: unadjusted; Model 2: adjusted for age, race, sex, energy; Model 3: adjusted for age, race, sex, energy, smoking status, physical activity, and weight gain/loss intentions; * *p* < 0.05, ^+^ *p* < 0.1.

## 4. Discussion

Findings from this analysis suggest that variability in dietary pattern adherence among freshmen attending a four-year university is associated with observable differences in select anthropometric and lipid biomarkers. Specifically, greater adherence to a Prudent dietary pattern, characterized by high consumption of plant-based foods, was favorably associated with body composition, while greater adherence to a dietary pattern characterized by higher, but modest, levels of alcohol consumption was favorably associated with blood lipid concentrations (mean intake less than one standard alcoholic beverage equivalents per day (0.95 standard drinks equaling 14 g pure alcohol)). Conversely, adherence to a Western dietary pattern had no impact on anthropometric outcomes but adverse impacts on lipid biomarkers among males.

Previous research supports Prudent dietary pattern adherence as protective and Western dietary pattern adherence as predisposing toward a variety of chronic disease outcomes, including type 2 diabetes, obesity, cancer, heart disease, anxiety/depression, and mortality among adults [41,53,54,55,56,57,58,59,60,61,62] and mental and metabolic health among adolescents [63,64,65,66]. Our findings extend the inverse associations between the Prudent pattern and anthropometric/biomarker indices to a younger population. Although previous empirically derived dietary pattern analyses in this population are lacking, our results are consistent with studies linking consumption of specific foods and food groups with health outcomes (e.g., Rose 2007 [67]).

A recent analysis of the Coronary Artery Risk Development in Young Adults (CARDIA) study data in young adults (18–30 years) reported a protective role of Mediterranean dietary pattern adherence against metabolic syndrome risk (abdominal obesity, elevated triglycerides, and low HDL cholesterol) in longitudinal models [68]. Although the CARDIA study was not restricted to young adults and employed researcher-defined dietary pattern parameters, similarities between the Mediterranean dietary pattern—rich in fruit, vegetables, whole grains, nuts, and fish and low in red and processed meat—in the aforementioned study and the Prudent pattern in ours are evident. Another study, investigating the relationship between nutrient intakes and adiposity in the National Heart, Lung, and Blood Institute Growth and Health Study cohort of 2371 black and white girls, (9–10 years of age at baseline) reported that the “healthy pattern”, characterized by high intake of fruit, vegetables, dairy, grains without added fats, mixed dishes and soups, and low intake of sweetened drinks, other sweets, fried foods, burgers, and pizza, was related to more favorable nutrient intakes and a smaller increase in waist circumference at age 19–20 years [69]. Although this population differed in age range and demographic profile, the findings were consistent with ours, since both studies supported a positive association between Prudent pattern adherence and adiposity outcomes among youth.

The deleterious association between the Western dietary patterndietary pattern score and cardiovascular disease biomarkers among males also agrees with previous research in adult populations [41,70]. Despite these adverse results, no associations were seen with adiposity outcomes among males in our study. Given the young age and generally healthy profile of our population, the lack of findings could suggest that adherence to the Western dietary pattern may affect lipid biomarkers of disease independently and/or prior to measurable changes in anthropometric outcomes in some cases, which may be important in early detection of cardiometabolic disease risk.

Interestingly, associations between the Western dietary pattern adherence and disease indicators in females were non-significant in adjusted models. Differences in the mean number of medium servings consumed from each food group by males and females scoring in the highest quartile of the Western dietary pattern (Table S2) may have accounted in part for the effect modification. It is also possible that biological differences in females (e.g., hormonal or neuro-endocrine differences) may mitigate or delay the adverse impacts seen in males. These hypotheses and other potential explanations merit further research.

Finally, the protective relationships observed between adherence to the Alcohol dietary pattern and lipid biomarkers are consistent with literature supporting a link between moderate consumption of alcohol and reduced risk for cardiovascular disease [71,72]. While previous studies have identified “alcohol“ or ”drinker“ patterns using empirically-derived dietary pattern methodology [73,74,75,76], the emergence of this pattern has generally been reported less consistently than variations of Prudent (healthy) and Western (unhealthy) patterns. However, increases in alcohol consumption during college has been observed, though its intake is likely to vary considerably within this population [77], which could help explain the emergence of a dietary pattern defined by alcohol consumption [78,79]. Nonetheless, even among students in the top quartile of dietary pattern adherence, average daily intake was relatively low (0.95 standard drinks). We did not observe significant associations between Alcohol dietary pattern adherence and anthropometric outcomes in our study, and few previous studies have looked specifically at alcohol consumption and overweight/obesity risk. However, several have considered relationships between alcohol consumption, especially binge drinking behavior, and eating patterns, body satisfaction, and weight loss intentions [80,81,82]. Findings from these studies suggest that alcohol abuse may be associated with disordered eating and/or lower diet quality. In one study, binge drinking was associated with poor diets, unhealthy weight control, body dissatisfaction, and sedentary behavior; although no independent associations between alcohol consumption and adiposity were reported, differences in alcohol-related eating behavior were associated with increased risk of overweight/obesity and, therefore, may moderate the association [83]. Students who “usually” or “always” ate before and/or during drinking were more likely to be overweight (RR: 1.24 (1.03–1.50)), with differences by year in school and weight status observed. In the general adult population a systematic review of previous studies did not support a positive association between alcohol consumption and adiposity [84]. Although positive findings between alcohol intake and weight gain have been reported, they have been primarily detected among high consumers; in contrast, some studies have suggested that light-to-moderate alcohol intake may protect against weight gain [84], which could also explain the lack of association seen here. In other studies, consumption of distilled alcoholic beverages has been positively associated with weight gain, whereas wine intake has shown a possible inverse association [84]. 

Future analyses should consider the frequency, amount, and type of alcohol consumed in relation to adiposity outcomes specifically among college students, as drinking patterns in this population differ from that of the general population [77,85,86]. It is also possible that alcohol consumption coincides with compensatory eating and/or exercise behaviors among college students [87]. Finally, though coffee was less strongly associated with the Alcohol pattern and mean intakes were relatively low (<6 fl oz per day), coffee consumption was found to be associated with increased total cholesterol, LDL cholesterol, and triglycerides in a systematic review of randomized trials [88], despite moderate consumption being consistently associated with reduced chronic disease risk in observational studies [89]. Further research linking beverage consumption and diet-related behavior could further clarify these relationships.

While findings from this study contribute to the scientific discussion on dietary patterns and health outcomes in an understudied age group, several limitations are apparent. First, the cross-sectional nature of the analysis limits our capacity to draw causal and temporal inferences from our findings. While models controlled for important variables hypothesized to confound or modify the relationship between dietary patterns and disease indicators, including total energy intake and physical activity, several relied upon self-report data, which are vulnerable to inherent biases. Moreover, it is possible that residual confounding could have affected the magnitude and/or significance of associations. For example, because dietary data were not collected for the time period prior to degree program matriculation, we could not account for the potential influence of childhood/adolescent dietary patterns on current dietary pattern and/or health status. Likewise, despite the relative sociodemographic homogeneity of our sample, we were not able to control for socioeconomic status in our models. Previous research suggests that income and education may influence diet quality among young adults [90] and, therefore, should be considered. We restricted our sample to freshman, nearly all of whom lived on campus and were required to purchase a University meal plan, regardless. Though previous literature on linking place of residence and dining habits with diet quality and health outcomes is mixed [23,91,92,93], results may or may not generalize to students living and/or dining off campus, which was more common among upperclassmen. The race/ethnicity of our sample was also predominantly non-Hispanic white, which may limit generalizability to more diverse populations.

Furthermore, it is possible that the variables that were measured and controlled in our analysis contain measurement error. Dietary intake was self-reported by FFQ and, consequently, subject to biases, such as recall error and observer/social desirability effects. Similarly, physical activity was assessed by self-report. However, the ACLS has been used in previous studies [42,43,44,94], and MET minutes were calculated to capture physical activity intensity and duration. We used the updated 2011 compendium, which included 800 unique activity-intensity levels [45], allowing for comprehensive and precise estimates of energy expenditure. Finally, although we relied on well-established methodology and conducted sensitivity analyses, PCA involves subjective decision-making (e.g., the total number of food groups included, number of patterns retained, pattern labeling, *etc.*), which could affect our results and their interpretation. For example, the percentage of the total variance explained by the derived factors is strongly influenced by the number of food groups included in the analysis and may also affect the precision of regression estimates [96,97,98]. Accordingly, we weighed eigenvalues and factor interpretability more heavily in deciding how many and which factors to retain for this analysis [98,99].

Despite these potential limitations, this study has notable strengths. In particular, the sample size was large (*n* = 683), even when limiting the analytic sample to students with complete data, including blood samples (*n* = 191). The comprehensiveness and measurement validity of our outcome variables, including measured height and weight, BIA-assessed body composition, and biomarkers collected via blood sample add merit to our findings. Finally, this study is one of few to evaluate associations between total diet and health status of first-year college students.

## 5. Conclusions

These results provide insight into potential associations between dietary patterns among college students and disease indices. The evidence could be strengthened by future studies using longitudinal methods to identify whether changes in dietary pattern adherence over multiple college years are associated with changes in anthropometric and biomarker measurements. It would also be of interest to identify predictors of dietary pattern choice. Understanding the relationship between diet and health among young adults attending colleges and universities is important for developing programs, policies, and behavior change strategies to improve quality of life and reduce diet-related disease burden at the population level.

## Supplementary Files

Supplementary File 1
