# Supplementary material for: Cross-Sectional Associations between Empirically-Derived Dietary Patterns and Indicators of Disease Risk among University Students
Source: Nutrients. 2015 Dec 24;8(1):3. doi: 10.3390/nu8010003 (PMC4728617; doi:10.3390/nu8010003)
Supplement: Supplementary File 1 [file nutrients-08-00003-s001.docx]

Supplementary Materials: Cross-Sectional Associations between Empirically-Derived
Dietary Patterns and Indicators of Disease Risk among University Students

Stacy A. Blondin ^1,^*, Megan P. Mueller ^1^, Peter J. Bakun ^1^, Silvina F. Choumenkovitch ^1^,
Katherine L. Tucker ^2^ and Christina D. Economos ^1^

**Table S1.** FFQ foods included in each food group used to derive dietary patterns via principal component analysis.

| **PCA Food Group Name** | **FFQ Foods Included** |
| --- | --- |
| Margarine | Margarine, 50% fat, Spread, HSoy/Cotn; Margarine, Reg, Stick/Tub, Unk; Margarine, Whipped |
| Butter | Butter, Regular |
| Other Fat and Oils | Lard, Salt Pork; Pam Spray; Gravy; Other Fats |
| Fruit | Banana; Apricot; Melons; Dried Fruit; Berries; Oranges; Grapefruits; Other Fruits; Other Citrus Fruits |
| Fruit Juice | Other Fruit Juices; Orange Juice; Grapefruit Juice |
| Breakfast Cereal | Other High Fiber Cereal; Cold Cereals, Regular; Granola Cereal; Fortified Cold Cereals |
| Whole Grains | Hot Cereals, Regular, Prepared; Wheat Bread, regular; Wheat Crackers; Oats/Bran Cereal |
| High Fat Dairy | Cottage Cheese, Regular; Cream, Half/Half; Cream, Heavy; Cheese, Regular; Whole Milk |
| Eggs | Eggs, Regular |
| Processed Meats | Processed Meat, Regular; Processed Meat, Extra Lean; Processed Meat, Lean |
| Legumes | Peas; Beans; Beans/Legumes Mixed Dishes |
| Other Vegetables | Corn; Other Vegetables; Allium (Onion, Shallots, Leeks |
| Tomatoes | Tomatoes; Vegetable Juice W/Vit C |
| Cruciferous Vegetables | Broccoli, Brussel Sprouts; Mustard Greens, Turnip Greens, Collards; Other Cruciferous (cauliflower; coleslaw ) |
| Dark Yellow and Orange Vegetables | Sweet Potato; Orange Vegetables; Mixed Vegetables; Winter Squash(butternut, acorn, pumpkin, spaghetti, hubbard) |
| Green Leafy Vegetables | Other Green Leafy Veg (Inc Spin; Lettuce (Iceberg Only)) |
| Creamy Dressing | Mayonnaise, Regular; Salad Dressing, Regular |
| Oil Dressings | Oils, Cottonseed |
| Diet Dressings | Imitation Mayonnaise, Regular; Salad Dressing, Lowfat |
| French Fries | Fried Potatoes, French Fries |
| Potatoes | Potatoes, Boil/Baked; Potato Salad; Mashed Potato |
| Refined Grains | White Bread, Regular; Biscuits; Rice, Plain; Muffins, Regular; Rolls; Corn Bread, muffins; Other Baked Goods, Regular; Tortilla, Flour; Crackers (regular; Tortilla, Corn) |
| Pasta | Pasta, Mixed dishes w/beef |
| Red Meat | Pork, Light; Pork, Regular; Beef, Xtra Light; Beef, Regular; Beef, Light; Beef Stew |
| Organ Meat | Liver, Fried; Liver, Regular |
| Poultry | Chicken/Turkey, Broil/Bake, W/Skin; Chicken/Turkey, Fried, W/Skin; Chicken/Turkey, Fried, WO/Skin; Chicken/Turkey, Broil/Bake, WO/Skin; Chicken/Turkey, Mixed Dishes |
| Fish and Shellfish | Fish, Fried; Tuna fish, Oil Pack; Shellfish; Fish, Regular; Tuna fish, Water Pack |
| Pizza | Pizza W/Meat |
| Non-creamy Soup | Homemade Soups, MW/Water; Veg & Tom Soups, MW/Water |
| Creamy Soup | Homemade Soups, Cream Based |
| Snacks | Snacks, Regular; Popcorn |
| Nuts | Nuts; Peanut Butter |
| Low Fat Dairy | Cheese, Lowfat; 2% Lowfat Milk; 1% Lowfat Milk; Ice Milk; Cottage Cheese, Lowfat; Skim Milk |
| Yogurt | Yogurt, Flavored; Yogurt, Regular |
| Sweets and Desserts | Gelatin; Sherbet; Frozen Yogurt, Regular; Sugars, syrups, jams; Sweets, Regular; Cakes, Regular; Cookies, Regular; Pies, Crisps, Cobblers; Custard |
| Ice Cream | Ice Cream, Regular |
| High Energy Drinks | Cola Caffeine free; Cola W/Caffeine |
| Low Energy Drinks | Diet Cola W/Caffeine |
| Beer | Beer, Regular |
| Wine | Wine, Red; Wine, White |
| Liquor | Liquor |
| Coffee | Coffee, Regular |
| Tea | Tea |

Notes: (1) Bacon, sausage, or hot dog, string beans, spreadable vegetables, mac and cheese, and coleslaw not included; (2) Dark yellow & orange veggies includes mixed veggies with carrots; French fries includes hash browns; low fat dairy includes low fat cheese; yogurt includes regular yogurt and flavored yogurt; sweets and desserts includes frozen yogurt; (3) Abbreviations: W: with; WO: without; MW: mixed with; Inc: include; Xtra: extra.

**Table S2.** Mean number of medium servings consumed per day among males and females with scores in the top quartile of the Western Dietary Pattern.

|  | **Males** | | **Females** | | **Difference** | **Significance** |
| --- | --- | --- | --- | --- | --- | --- |
| **PCA Food Group** | **Mean** | **SD** | **Mean** | **SD** |  |  |
| Beer | 0.39 | 0.54 | 0.14 | 0.23 | 0.25 | **** |
| Breakfast cereal | 0.70 | 0.72 | 0.55 | 0.51 | 0.15 |  |
| Butter ^†^ | 0.33 | 0.39 | 0.45 | 0.48 | −0.12 | * |
| Coffee | 0.16 | 0.41 | 0.35 | 0.88 | −0.19 | * |
| Cream-based soup | 0.07 | 0.11 | 0.11 | 0.16 | −0.04 | * |
| Creamy dressing ^†^ | 0.31 | 0.27 | 0.28 | 0.24 | 0.03 |  |
| Cruciferous vegetables | 0.20 | 0.25 | 0.22 | 0.26 | −0.02 |  |
| Diet dressing | 0.27 | 0.29 | 0.29 | 0.29 | −0.02 |  |
| Dark yellow/orange vegetables | 0.27 | 0.29 | 0.25 | 0.31 | 0.02 |  |
| Eggs | 0.31 | 0.32 | 0.2 | 0.24 | 0.11 | ** |
| Fish | 0.26 | 0.38 | 0.28 | 0.33 | −0.02 |  |
| French fries ^‡^ | 0.98 | 0.72 | 0.80 | 0.58 | 0.18 | * |
| Fruit | 1.20 | 0.86 | 1.10 | 0.89 | 0.10 |  |
| Fruit juice ^†^ | 2.05 | 1.66 | 1.61 | 1.53 | 0.44 | * |
| Green leafy vegetables | 0.68 | 0.52 | 0.58 | 0.47 | 0.10 |  |
| High fat dairy ^†^ | 0.91 | 0.85 | 0.72 | 0.69 | 0.19 |  |
| High energy drinks ^†^ | 0.95 | 1.11 | 0.37 | 0.67 | 0.58 | **** |
| Ice cream ^†^ | 0.33 | 0.31 | 0.34 | 0.31 | −0.01 |  |
| Legumes | 0.17 | 0.19 | 0.11 | 0.12 | 0.06 | ** |
| Liquor | 0.25 | 0.31 | 0.19 | 0.41 | 0.06 |  |
| Low fat dairy | 2.00 | 2.31 | 1.37 | 1.55 | 0.63 | * |
| Low energy drinks | 0.26 | 0.96 | 0.20 | 0.61 | 0.06 |  |
| Margarine ^†^ | 0.24 | 0.36 | 0.39 | 0.48 | −0.15 | ** |
| Non-creamy soup | 0.12 | 0.16 | 0.18 | 0.23 | −0.06 | * |
| Nuts | 0.18 | 0.25 | 0.20 | 0.30 | −0.02 |  |
| Oil-based dressing | 0.15 | 0.29 | 0.28 | 0.49 | −0.13 | * |
| Organ meats | 0.01 | 0.05 | 0.00 | 0.01 | 0.01 | * |
| Other fats and oils ^†^ | 0.13 | 0.19 | 0.18 | 0.26 | −0.05 |  |
| Other vegetables | 0.33 | 0.37 | 0.27 | 0.32 | 0.06 |  |
| Pasta ^†^ | 0.74 | 0.55 | 0.67 | 0.46 | 0.07 |  |
| Pizza ^†^ | 0.53 | 0.35 | 0.37 | 0.31 | 0.16 | *** |
| Potatoes ^†^ | 0.29 | 0.26 | 0.35 | 0.29 | −0.06 |  |
| Poultry | 0.69 | 0.59 | 0.63 | 0.43 | 0.06 |  |
| Processed meats ^‡^ | 0.65 | 0.54 | 0.34 | 0.36 | 0.31 | **** |
| Red meat ^‡^ | 0.80 | 0.53 | 0.42 | 0.44 | 0.38 | **** |
| Refined grains ^‡^ | 1.93 | 1.17 | 1.84 | 0.98 | 0.09 |  |
| Snacks ^‡^ | 0.33 | 0.35 | 0.32 | 0.28 | 0.01 |  |
| Sweets and desserts ^†^ | 1.15 | 0.86 | 1.72 | 1.50 | −0.57 | *** |
| Tea | 0.28 | 0.65 | 0.29 | 0.43 | −0.01 |  |
| Tomatoes | 0.15 | 0.23 | 0.16 | 0.28 | −0.01 |  |
| Whole grains | 0.40 | 0.52 | 0.41 | 0.47 | −0.01 |  |
| Wine | 0.04 | 0.10 | 0.03 | 0.08 | 0.01 |  |
| Yogurt | 0.18 | 0.36 | 0.27 | 0.40 | −0.09 |  |

* <0.05; ** <0.01; *** <0.001; *** <0.0001; † indicates food groups with factor loading >0.3; ‡ indicates food groups with factor loadings >0.5.

**Figure S1.** Scree plot of eigenvalues resulting from principal component analysis.
